# Combined inhibition of atrial‐specific potassium currents as a possible anti‐arrhythmic drug therapy: A match made *in silico*

**DOI:** 10.1113/JP287950

**Published:** 2024-12-13

**Authors:** Vladimír Sobota

**Affiliations:** ^1^ IHU Liryc, Electrophysiology and Heart Modeling Institute Fondation Bordeaux Université Bordeaux France; ^2^ Institut de Mathématiques de Bordeaux University of Bordeaux Talence France

**Keywords:** anti‐arrhythmic drugs, atrial fibrillation, computational models, *in silico* trials

Atrial fibrillation (AF) represents a major challenge for public health and a considerable socioeconomic burden. Anti‐arrhythmic therapy that aims to restore and maintain sinus rhythm, together with anti‐coagulation therapy to prevent AF‐related stroke, form the cornerstones of AF management (Van Gelder et al., 2024). Several options are available for rhythm‐control therapy, including catheter ablation, electrical cardioversion, and pharmacological therapy. Unfortunately, none of these therapeutic options provide satisfying outcomes, especially over the long term. This creates an unmet need for novel therapeutic strategies that would improve AF management, and for methods that can facilitate their identification and development.

The use of currently available anti‐arrhythmic drugs (AADs) for treatment of AF is severely limited by their side effects, particularly by proarrhythmic effects in the ventricles. This restricts the highest doses that can be safely administered and constrains the therapeutic potential of AADs. To overcome this major drawback, the focus has shifted towards atrial‐specific AADs that target ion channels predominantly expressed in human atria. Several atrial‐specific ion channels have been identified, including small‐conductance calcium‐activated potassium channels (SK) and two‐pore domain potassium channels (K2P). Both the SK current (*I*_SK_) and TASK‐1, a member of the K2P current family (*I*_K2P_), contribute to atrial repolarization and have been found upregulated in AF patients (Wiedmann & Schmidt, 2024), making them attractive targets for AAD therapy.

In this issue of *The Journal of Physiology*, Dasí et al. (2026) present a prospective *in silico* trial in which they evaluate the efficacy of single *I*_SK_ and *I*_K2P_ inhibition, and of their combination, to terminate AF (Dasí et al., 2026). The study utilized 1000 atrial models created by combining different bi‐atrial anatomies, cardiomyocyte models that capture the variability in ionic current densities observed in AF patients, and structural heterogeneities characterized by low‐voltage areas. In 654 of these models, sustained AF was observed, and these models were subjected to the simulated anti‐arrhythmic pharmacotherapies. Although single *I*_SK_ and *I*_K2P_ inhibition resulted in cardioversion rates of 33% and 43%, respectively, inhibition of both currents terminated AF in 82% of atrial models. The combined therapy successfully cardioverted AF in 175 models (27% of all models with sustained AF) that did not respond to either single ion channel inhibition therapy, demonstrating synergistic effect of the combined approach.

Investigating the results further, Dasí et al. (2026) observed that the simulated pharmacological therapies remained unsuccessful in the atria that exhibited short refractoriness due to upregulated repolarization currents. By contrast, most of the models in which *I*_K2P_ inhibition led to AF termination also exhibited low *I*_SK_ density and, similarly, the majority of cardioversions caused by *I*_SK_ inhibition took place in models with low *I*_K2P_ density. These findings indicate that AF termination through inhibition of a single ion channel may be effective only in some arrhythmogenic substrates, and that higher cardioversion efficacy probably requires a multitarget approach.

Obviously, the study presents an ideal scenario in which complete *I*_SK_ and *I*_K2P_ inhibition can be achieved, which is rarely the case in clinically available AADs. Although the cardioversion efficacies reported in the study could be seen as the highest theoretically achievable, it does not mean these outcomes are unrealistic. Recent phase 2 clinical trial that investigated selective *I*_SK_ inhibitor AP30663 reported cardioversion efficacies of 42% and 55% for the doses of 3 and 5 mg/kg, respectively (Holst et al., 2024). These cardioversion rates are even higher than the one predicted by Dasí et al. (2026) (33%), probably as a result of an off‐target inhibition of the K_v_11.1 channel. It will be therefore interesting to compare the results of *I*_K2P_ inhibition predicted by this *in silico* trial with the future outcome of the DOCTOS trial that investigates the efficacy of TASK‐1 inhibitor doxapram to terminate AF (EudraCT No 2018‐002979‐17).

Taken together, the study by Dasí et al. (2026) illustrates how *in silico* trials can help to identify new anti‐arrhythmic drug therapies. Building on previous *in silico* findings from atrial myocyte models (Ni et al., 2020), this work takes an important step forward by testing the combined potassium current inhibition in computational models of human atria and estimating its cardioversion efficacy, bringing the approach closer to the real‐world clinical setting. It will be compelling to see how the outcomes of future clinical trials assessing anti‐arrhythmic effects of *I*_SK_ and *I*_K2P_ inhibitors, and possibly their combined use, align with these findings, providing an ultimate test to the predictive value of this *in silico* trial.

## Additional information

### Competing interests

No competing interests declared.

### Author contributions

Sole author.

### Funding

The author's research is supported by Fondation Lefoulon‐Delalande and by the French National Research Agency grant ANR‐10‐IAHU‐04.

## Supporting information


Peer Review History
