# Supplementary material for: Combined inhibition of atrial‐specific potassium currents as a possible anti‐arrhythmic drug therapy: A match made in silico
Source: J Physiol. 2024 Dec 13;604(13):5088–9. doi: 10.1113/JP287950 (PMC13327749; doi:10.1113/JP287950)
Supplement: Supplementary file 1 — Peer Review History [file TJP-604-5088-s001.pdf]

The Journal of Physiology

Combined inhibition of atrial-specific potassium currents as a possible anti-arrhythmic drug therapy: a match made in silico

Vladimír Sobota
DOI: 10.1113/JP287950

Corresponding author(s): Vladimír Sobota (vladimir.sobota@ihu-liryc.fr)

The referees have opted to remain anonymous.

**Review Timeline:**	Submission Date:	04-Nov-2024
	Accepted:	28-Nov-2024

Senior Editor: Bjorn Knollmann

Reviewing Editor: Viviane Timmermann

Transaction Report:

(Note: With the exception of the correction of typographical or spelling errors that could be a source of ambiguity, letters and reports are not edited. Depending on transfer agreements, referee reports obtained elsewhere may or may not be included in this compilation. Referee reports are anonymous unless the Referee chooses to sign their reports.)

Dear Dr Sobota,

Re: JP-TP-2024-287950 "Combined inhibition of atrial-specific potassium currents as a possible anti-arrhythmic drug therapy: a match made in silico" by Vladimír Sobota

We are pleased to tell you that your paper has been accepted for publication in The Journal of Physiology.

TRANSPARENT PEER REVIEW POLICY: To improve the transparency of its peer review process, The Journal of Physiology publishes online as supporting information the peer review history of all articles accepted for publication. Readers will have access to decision letters, including Editors' comments and referee reports, for each version of the manuscript, as well as any author responses to peer review comments. Referees can decide whether or not they wish to be named on the peer review history document.

The last Word (or similar) version of the manuscript provided will be used by the Production Editor to prepare your proof. When this is ready you will receive an email containing a link to Wiley's Online Proofing System. The proof should be thoroughly checked and corrected as promptly as possible.

Authors should note that it is too late at this point to offer corrections prior to proofing. Major corrections at proof stage, such as changes to figures, will be referred to the Editors for approval before they can be incorporated. Only minor changes, such as to style and consistency, should be made at proof stage. Changes that need to be made after proof stage will usually require a formal correction notice.

All queries at proof stage should be sent to: TJP@wiley.com.

Are you on Twitter? Once your paper is online, why not share your achievement with your followers? Please tag The Journal (@jphysiol) in any tweets and we will share your accepted paper with our 30,000 followers!

If you would like to receive our 'Research Roundup', a monthly newsletter highlighting the cutting-edge research published in The Physiological Society's family of journals (The Journal of Physiology, Experimental Physiology and Physiological Reports), please click this link, fill in your name and email address and select 'Research Roundup':
https://www.physoc.org/journals-and-media/membernews/

Yours sincerely,

Bjorn Knollmann
Senior Editor
The Journal of Physiology

EDITOR COMMENTS

Reviewing Editor:

Comments to the Author:
Thank you for reviewing the editorial. I agree that the author did a very good job in writing this editorial.

Senior Editor:

Comments to the Author:
Thank you for this well-written and informative editorial!

REVIEWER COMMENTS

Referee #1:

I, on behalf of the authors, express our gratitude to Vladimír Sobota.

This is a very nice editorial, with a significant appraisal of the methodological design of our study.

P.S. - You can help your research get the attention it deserves! Check out Wiley's free Promotion Guide for best-practice recommendations for promoting your work at www.wileyauthors.com/eeo/guide. You can learn more about Wiley Editing Services which offers professional video, design, and writing services to create shareable video abstracts, infographics, conference posters, lay summaries, and research news stories for your research at www.wileyauthors.com/eeo/promotion.

IMPORTANT NOTICE ABOUT OPEN ACCESS: To assist authors whose funding agencies mandate public access to published research findings sooner than 12 months after publication, The Journal of Physiology allows authors to pay an Open Access (OA) fee to have their papers made freely available immediately on publication.

The Corresponding Author will receive an email from Wiley with details on how to register or log-in to Wiley Authors Services where you will be able to place an order.

You can check if your funder or institution has a Wiley Open Access Account here: https://authorservices.wiley.com/author-resources/Journal-Authors/licensing-and-open-access/open-access/author-compliance-tool.html.
